# STAT6 deficiency ameliorates Graves' disease severity by suppressing thyroid epithelial cell hyperplasia

**DOI:** 10.1038/cddis.2016.398

**Published:** 2016-12-01

**Authors:** Xuechao Jiang, Bingbing Zha, Xiaoming Liu, Ronghua Liu, Jun Liu, Enyu Huang, Tingting Qian, Jiajing Liu, Zhiming Wang, Dan Zhang, Luman Wang, Yiwei Chu

**Affiliations:** 1 Department of Immunology and Key Laboratory of Medical Molecular Virology of MOE/MOH, School of Basic Medical Sciences, Biotherapy Research Center of Fudan University, Shanghai, 200032, China; 2 Scientific Research Center, Xinhua Hospital, Shanghai JiaoTong University School of Medicine, Shanghai, PR China; 3 Department of Endocrinology and Metabolism, Shanghai Fifth People's Hospital, Fudan University, Shanghai, China

## Abstract

Signal transducer and activator of transcription 6 (STAT6) is involved in epithelial cell growth. However, little is known regarding the STAT6 phosphorylation status in Graves' disease (GD) and its role in thyroid epithelial cells (TECs). In this study, we found that STAT6 phosphorylation (p-STAT6) was significantly increased in TECs from both GD patients and experimental autoimmune Graves' disease mice and that STAT6 deficiency ameliorated GD symptoms. Autocrine IL-4 signalling in TECs activated the phosphorylation of STAT6 via IL-4 R engagement, and the downstream targets of STAT6 were Bcl-xL and cyclin D1. Thus, the IL-4-STAT6-Bcl-xL/cyclin D1 pathway is crucial for TEC hyperplasia, which aggravates GD. More importantly, *in vitro* and *in vivo* experiments demonstrated that STAT6 phosphorylation inhibited by AS1517499 decreased TEC hyperplasia, thereby reducing serum T3 and T4 and ameliorating GD. Thus, our study reveals that in addition to the traditional pathogenesis of GD, in which autoantibody TRAb stimulates thyroid-stimulating hormone receptors and consequently produces T3, T4, TRAb could also trigger TECs producing IL-4, and IL-4 then acts in an autocrine manner to activate p-STAT6 signalling and stimulate unrestricted cell growth, thus aggravating GD. These findings suggest that STAT6 inhibitors could be potent therapeutics for treating GD.

Graves' disease (GD), a common organ-specific autoimmune disease, usually results in hyperthyroidism (80–85% of GD cases), which is accompanied by a series of pathophysiological symptoms including irritability, muscle weakness, sleeping problems, rapid heartbeat, poor tolerance of heat, diarrhoea and weight loss.^1^ GD affects approximately 1–2% of people worldwide, and its incidence is increasing.^2^ Treatments for GD include antithyroid drugs, radioiodine and thyroidectomy, which reduce the production of thyroid hormone by destroying or removing the thyroid tissue.^3^ However, due to poor understanding of the exact aetiology of GD, the treatments are often invasive and ineffective, and they have not changed over the past 50 years.^3^ Thus, a better understanding of the key molecules and mechanisms that mediate the pathogenesis of GD is of great theoretical and practical significance.

Macroscopically, GD and its most severe form, hyperthyroidism, are typically characterized by thyromegaly.^4^ Microscopically, histological thyroid epithelial cell (TEC) hyperplasia is the salient criterion of GD diagnosis.^1^ Although the aetiology of GD remains unclear, one widely accepted mechanism of pathogenesis is that anti-TSH receptor autoantibodies (TRAbs) promote TEC growth and unrestricted thyroid hormone T3 and T4 secretion by TSH mimics.^5, 6^ However, treatment using rituximab to deplete TRAbs causes many side effects, such as aggravating ulcerative colitis.^7, 8^ Thus, we believe that in addition to TRAb depletion, there may be an alternative treatment for the TEC hyperplasia associated with GD.

Signal transducer and activator of transcription 6 (STAT6) is a critical transcription factor in cytokine production and polarization of immune cells.^9, 10^ Activation of STAT6 has also been suggested to promote epithelial cell growth in the lung, skin and intestine.^11, 12, 13^ STAT6 has been reported to be an aggravating factor in GD; however, the exact mechanisms are still unclear.^35^ Cytokines, such as IL-4, IL-13 and IL-22 are important for supporting and maintaining antibody-mediated immune responses in GD, and they are also potent triggers of the phosphorylation of STAT6.^14, 15, 16, 17^ Phosphorylated STAT6 dimerizes and translocates to the nucleus to activate target genes involved in cell proliferation, such as *Bcl-2, Bcl-xl, pcna* and *cyclin d1*.^18, 19, 20, 21, 22^ However, the precise molecular pathway of STAT6-dependent excessive proliferation is not clear.

In the present study, we aimed to determine whether STAT6 has a crucial role in TEC hyperplasia during GD. To address this question, we first showed that STAT6 phosphorylation (p-STAT6) was upregulated in TECs of GD patients. We investigated STAT6-dependent TEC hyperplasia mechanisms and found that TEC-derived IL-4 triggered the phosphorylation of STAT6, and p-STAT6 then induced high expression levels of Bcl-xL and cyclin D1 during GD, which resulted in TEC hyperplasia. Administering AS1517499, a STAT6 phosphorylation inhibitor, to experimental autoimmune Graves' disease (EAGD) mice resulted in marked amelioration of disease signs.

## Results

### STAT6 phosphorylation increases in TECs from both GD patients and EAGD mice

**Figure 1:**
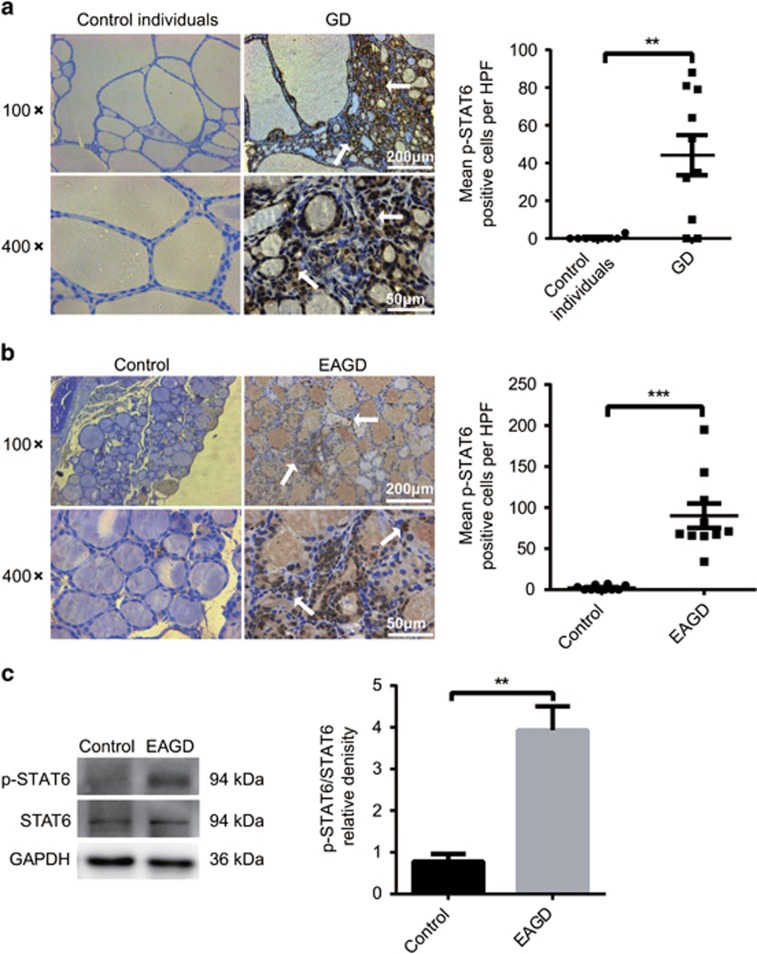
p-STAT6 was increased in TECs from both GD patients and EAGD mice. (**a**) p-STAT6 was measured in eight control individuals and 10 GD patients by IHC staining. Representative p-STAT6 staining in control individuals and GD patients. Arrow points to a p-STAT6-positive TEC. Magnification: × 100; × 400 (left panel). IHC was blindly scored by counting the positive cells in 10 HPFs, and individual thyroid gland scores are shown, with each point representing a single person (right panel). (**b**) p-STAT6 was measured in 10 control mice and 10 EAGD mice by IHC staining. Representative p-STAT6 staining in control mice and EAGD mice. Arrow points to a p-STAT6-positive TEC. Magnification: × 100; × 400 (left panel). IHC was blindly scored by counting the positive cells in 10 HPFs, and individual thyroid gland scores are shown, with each point representing one mouse (right panel). (**c**) Protein was isolated from individual fresh thyroid glands, and p-STAT6 protein was measured by western blotting. GAPDH was used as a loading control. Immunoblot analysis of p-STAT6 in the left panel with the relative band densities shown in the right panel. We analysed more than three individual mouse thyroid glands in every experiment. Independent experiments were repeated four times, and a representative figure is shown. The data shown are the means±S.E.M. of one experiment with 10 mice. Three repetitions showed similar results. Scale bars (× 100): 200 μm; scale bars (× 400): 50 μm. **P<0.01 and ***P<0.001. Columns and error bars represent the means±S.E.M.

Thyroid TEC hyperplasia is a typical feature found during histological examination of GD tissue. p-STAT6 is closely related to epithelial cell hyperplasia, which is the cause of ulcerative colitis, lung inflammation and skin inflammation.^11, 12, 13^ To investigate whether p-STAT6 plays a similar role in GD, we first explored p-STAT6 expression in TECs from GD patients and control individuals. Immunohistochemical staining revealed that p-STAT6 was highly expressed in TECs of GD patients compared with cells of control individuals (Figure 1a). Because *in vivo* experiments cannot be performed in humans, we used a widely accepted GD mouse model called the EAGD mouse model, which was induced by repeatedly immunizing mice with an adenovirus vector expressing TSHR-289. TSH-binding inhibition (TBI), T3 and T4 in mouse serum were measured 4 weeks after three injections of Ad-TSHR289. As shown in Supplementary Figure 1, the EAGD mouse model was successfully generated. The details are provided in the figure legend. It is noteworthy that histologically, TECs exhibited hyperplasia and hypercellularity with intrusion into the follicular lumen, which is highly consistent with the clinical features of GD (Supplementary Figure S1F). To confirm the expression of p-STAT6 in mouse TECs, we analysed the p-STAT6 level using immunohistochemistry (IHC) and western blot analysis in both the EAGD group and control group. We found that the p-STAT6 level was markedly increased in the TECs of EAGD mice compared with control mice, according to both IHC and western blot analysis (Figures 1b and c). Together, our results demonstrate that STAT6 phosphorylation was significantly increased in TECs from both GD patients and EAGD mice.

### STAT6 deficiency ameliorates GD

**Figure 2:**
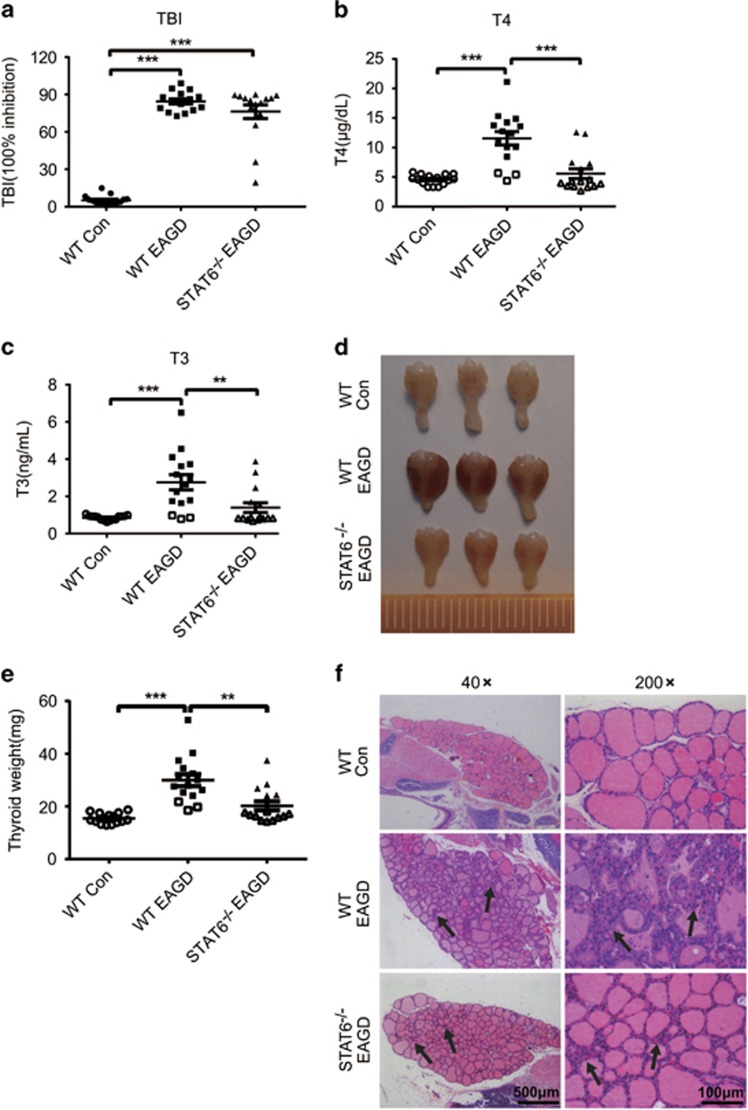
STAT6 deficiency ameliorated EAGD. EAGD was generated in WT and STAT6^−/−^ mice by injection with TSHR-289-expressing adenovirus as shown in Supplementary Figure 1. Mice were euthanized 4 weeks after three injections, and blood and thyroid glands were obtained. Sera were analysed for TBI (**a**), T4 (**b**) and T3 (**c**), and data for individual mice are shown. (**d**) Thyroid glands were harvested for gross comparison and weight determination. Representative images show the appearance of thyroid glands from the WT control, WT EAGD and STAT6^−/−^ EAGD groups. (**e**) The weights of thyroids were measured (left panel). Individual weights of thyroids are shown with each point. (**f**) Representative histological sections of thyroid glands stained with H&E are shown for the WT control, WT EAGD and STAT6^−/−^ EAGD groups. Images are shown at × 40 and × 200 magnifications. An arrow indicates the position of TEC hyperplasia in the histological images. The data shown are the means±S.E.M. of one experiment with 15 mice. Three independent experiments showed similar results. Scale bars (× 40): 500 μm; scale bars (× 200): 100 μm. **P<0.01 and ***P<0.001

Hypothetically, STAT6 regulation of TECs is a critical factor in GD pathology; thus, STAT6 depletion should ameliorate GD. Therefore, we induced GD in STAT6^−/−^ mice. As shown in Figure 2, compared with wild-type (WT) mice, STAT6^−/−^ mice injected with Ad-TSHR-289 exhibited significantly lower serum T3 and T4 levels and reduced disease severity based on thyroid appearance and weight. The size and weight of the thyroid glands were similar to those of the thyroid glands in the control mice. Histologically, TECs in the WT EAGD mice were characterized by more columnar and vacuolated hyperplastic cells. However, these cell and tissue changes were decreased in the STAT6^−/−^ mice. Thus, it is highly likely that STAT6 is involved in TEC hyperplasia in GD.

To exclude the possibility that *stat6* gene deficiency spontaneously induced GD, we compared the WT Con and STAT6^−/−^ Con groups. As shown in Supplementary Figure 2, the STAT6^−/−^ Con mice exhibited similar T3 and T4 levels and histological features, as well as a similar thyroid appearance, to the WT Con mice, indicating that *stat6* gene deficiency does not affect the function of the thyroid. Moreover, to investigate any perturbation in GD-related cytokine production and antibody isotype switching, the serum from mice was tested for IL-4, IgG1 and IgG2a. As shown in Supplementary Figures 3A, C and E, STAT6^−/−^ Con mice expressed parallel IL-4, IgG1 and IgG2a levels compared with WT Con mice, as did STAT6^−/−^ EAGD mice versus WT EAGD mice. These results demonstrated that the Th2 differentiation and Ig class switching were unaffected by STAT6 deficiency.

### STAT6 promotes hyperplasia of TECs by upregulating Bcl-xL and cyclin D1

**Figure 3:**
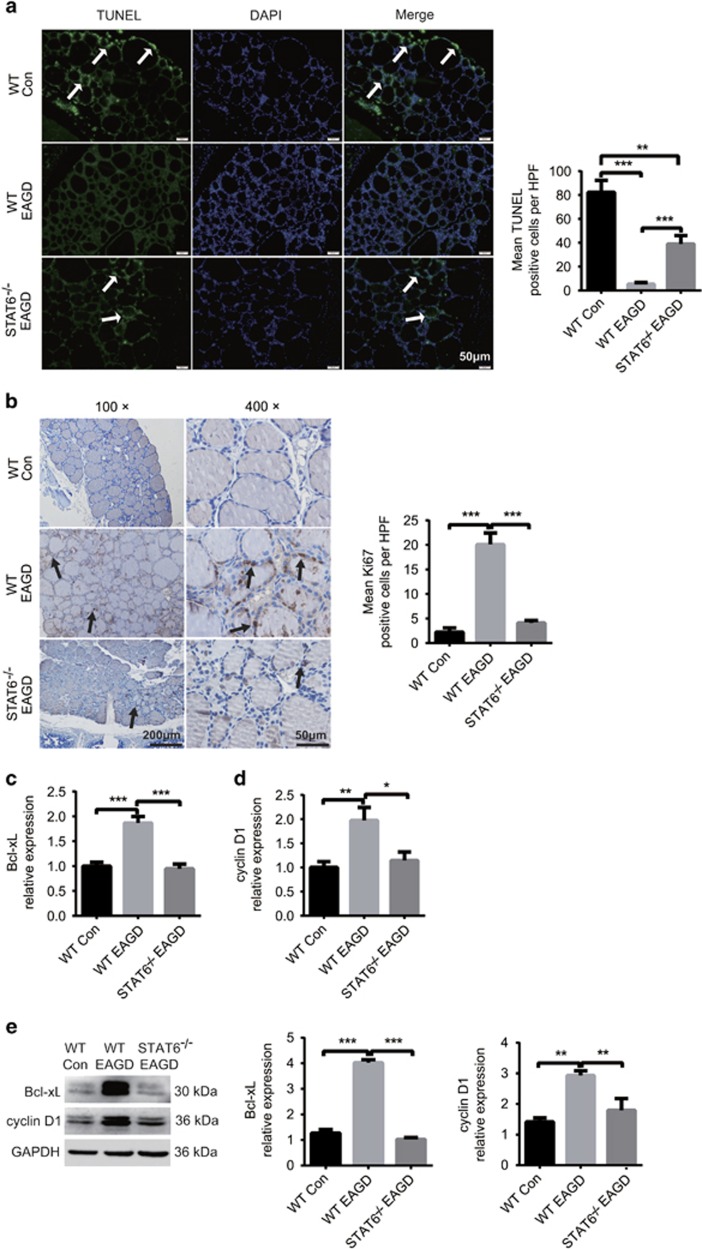
STAT6 promoted TEC growth by inducing the expression of Bcl-xL and cyclin D1 EAGD was generated in WT and STAT6^−/−^ mice by injection with TSHR-289-expressing adenovirus as shown in Supplementary Figure 1. Mice were euthanized 4 weeks after three injections, and the thyroid glands were obtained. IF and IHC were used to assess the growth of TECs in the different groups. (**a**) Thyroid gland sections from WT and STAT6^−/−^ mice were assayed for TUNEL positivity (green) and stained with DAPI (blue) to determine the degree of TEC apoptosis. Apoptosis was measured in eight mice from each group. Representative staining is shown in the left panel. Arrows point to TUNEL-positive TECs. Magnification: × 200. IF was blindly scored by counting positive cells in 10 HPFs (right panel). (**b**) Ki67 was also measured in eight mice from each group by IHC staining. Representative Ki67 staining in each group is shown in the left panel. Arrow points to Ki67-positive TECs. Magnification: × 100; × 400 (left panel). IHC was blindly scored by counting positive cells in 10 HPFs (right panel). (**c**) Individual thyroid glands were obtained and homogenized. RNA and protein were then extracted from the homogenates. The mRNA expression of Bcl-xL (**c**) and cyclin D1 (**d**) was examined by quantitative real-time PCR analysis. (**e**) Bcl-xL and cyclin D1 protein levels were measured by western blotting. Immunoblot analysis of Bcl-xL and cyclin D1 levels in the individual thyroid gland were obtained from their relative band densities. GAPDH was used as the loading control. We analysed more than three individual mouse thyroid glands in every experiment. Independent experiments were repeated four times and a representative figure is shown. The data shown are the means±S.E.M. of one experiment with eight mice. Three replicates showed similar results. Scale bars in A: (× 200): 50 μm. Scale bars in B (× 100): 200 μm; scale bars (× 400): 50 μm. *P<0.05, **P<0.01 and ***P<0.001

STAT6 is involved in cell proliferation and prevents cell apoptosis, which could result in cellular hyperplasia and hypercellularity. As shown in Figures 3a and b, TECs from the WT EAGD group showed relatively high Ki67 and low TUNEL staining, which demonstrated that TECs proliferated vigorously and apoptosed at a low rate in GD. In contrast, TECs from the STAT6^−/−^ EAGD group showed relatively low Ki67 and high TUNEL staining, which meant that STAT6 was able to promote TEC hyperplasia.

Expression of anti-apoptotic and pro-proliferation proteins is an important mechanism for vigorous growth of epithelial cells.^23, 24^ We therefore sought to investigate which candidate genes in TECs are upregulated in GD and, more importantly, whether this induction is mediated by STAT6. We first performed real-time RT-PCR to evaluate the mRNA levels of *bcl-xl, bcl-2, pcna, cyclin d1*, and other genes in TECs from both WT and STAT6^−/−^ EAGD mice. We found that *bcl-xl* and *cyclin d1* were strongly upregulated in TECs from WT EAGD mice and were significantly downregulated to control levels in TECs from the STAT6^−/−^ EAGD mice (Figures 3c and d). We confirmed the results at the protein level by western blot analysis. The Bcl-xL and cyclin D1 levels in TECs from WT EAGD mice were more than twofold greater than those in the TECs from WT control mice, while the levels in TECs from the STAT6^−/−^ EAGD mice were significantly decreased, similar to the mRNA levels (Figure 3e). These data suggest that Bcl-xL and cyclin D1 are highly induced in TECs in GD and are regulated by STAT6.

### STAT6 phosphorylation in TECs is activated by IL-4

**Figure 4:**
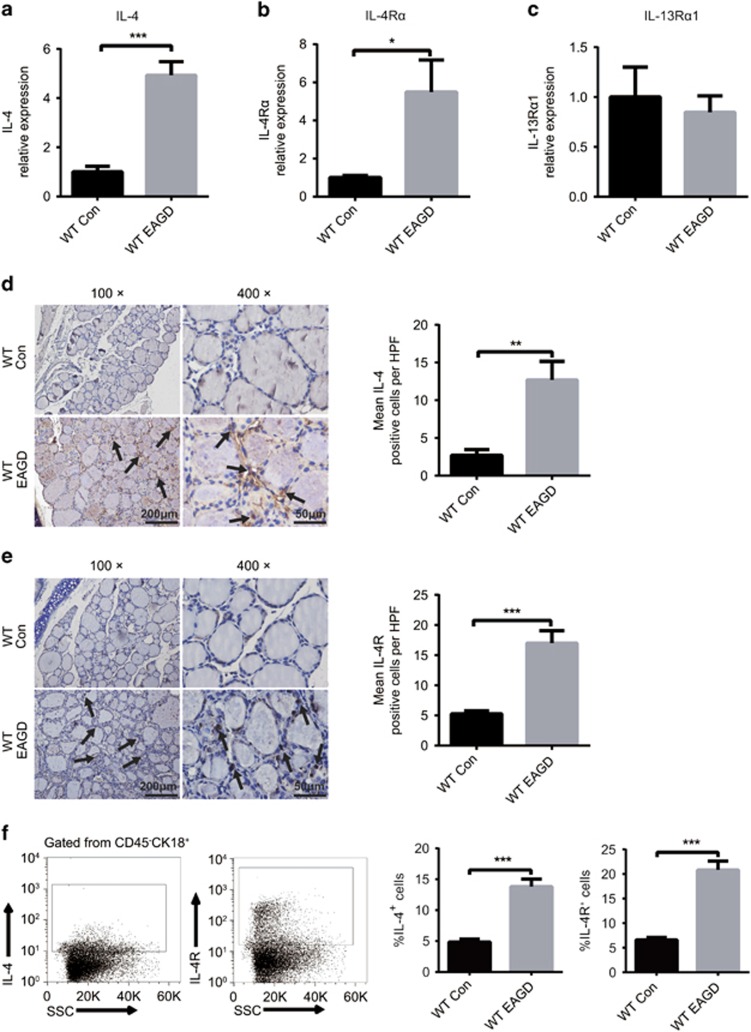
The expression of IL-4 and IL-4 receptors in the thyroid of control and EAGD mice. Thyroid glands were obtained from control and EAGD mice 4 weeks after three injections. Quantitative real-time PCR analysis of IL-4 (**a**), IL-4 R*α* (**b**) and IL-13R*α*1 (**c**) in fresh thyroid glands. (**d**) IL-4 protein was measured in 10 control mice and 10 EAGD mice by IHC straining. Representative IL-4 staining in control mice and EAGD mice. The arrow points to IL-4-positive thyroid follicular epithelial cells. Magnification: × 100; × 400 (left panel). IHC was blindly scored by counting positive cells in 10 HPFs, and individual thyroid gland scores are shown (right panel). (**e**) IL-4 R protein was measured in 10 control mice and 10 EAGD mice by IHC straining. Representative IL-4 R staining in control mice and EAGD mice. The arrow points to IL-4 R-positive thyroid follicular epithelial cells. Magnification: × 100; × 400 (left panel). IHC was blindly scored by counting positive cells in 10 HPFs, and individual thyroid gland scores are shown, with each point representing one mouse (right panel). Three repetitions showed similar results. Scale bars (× 100): 200 μm; scale bars (× 400): 50 μm. (**f**) Gating strategy for the identification of IL-4^+^ and IL-4 R^+^ TECs (left panel), and percentages of IL-4^+^ and IL-4 R^+^ TECs are shown (right panel). *P<0.05, **P<0.01 and ***P<0.001

To further explain why p-STAT6 was unregulated in GD, we determined the factors that activated STAT6 in TECs in GD. IL-4, IL-13, IL-22, IL-25 and IL-33 are potent upstream activators of STAT6. Thus, we first used real-time RT-PCR to evaluate these cytokines in both the control and EAGD mice. As shown in Figures 4a–c and Supplementary Figure 4, the control and EAGD mice exhibited comparable mRNA levels of *il-4, il-13, il-22, il-25 and il-33*, none of which were upregulated in the thyroids of EAGD mice except *il-4* and its receptor, which demonstrated that IL-4 production increased in the EAGD mice and could be important in GD. To confirm these results, we used IHC and fluorescence-activated cell sorter (FACS) to detect IL-4 protein in control and EAGD mice. We found that IL-4 production in TECs from EAGD mice was significantly increased. Surprisingly, we also found that thyroid-infiltrating IL-4 was derived from the TECs themselves, and the TECs highly expressed IL-4 R (Figures 4d–f), which demonstrated that TECs could secrete IL-4 and stimulate their own hyperplasia through an autocrine mechanism.

**Figure 5:**
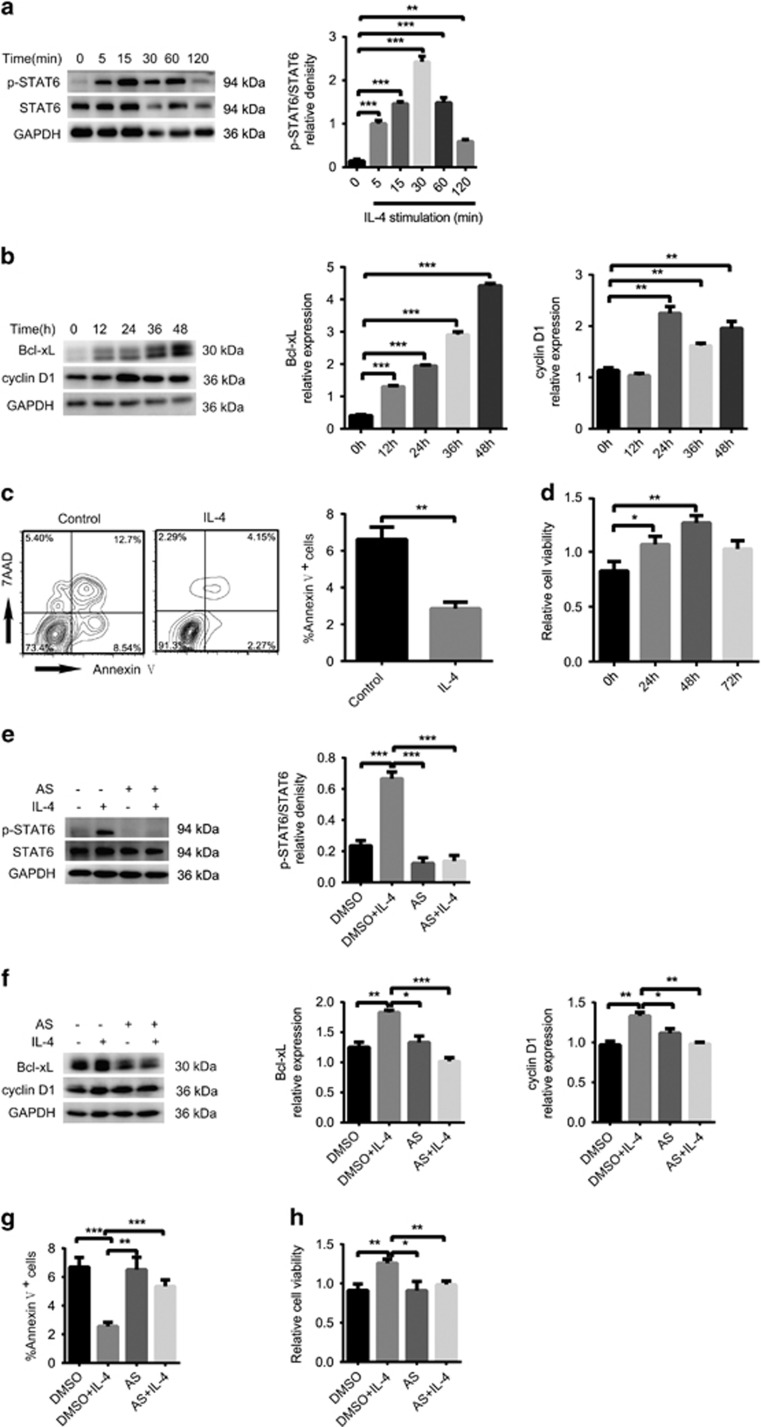
Phosphorylation of STAT6 is activated by IL-4 Nthy-ori 3-1 cells were stimulated by IL-4, and cells were harvested at different time points. (**a**) p-STAT6 was measured by western blotting (left panel). Immunoblot analysis of the ratio of pSTAT6/STAT6 in Nthy-ori 3-1 cells with their relative band densities (right panel). (**b**) Bcl-xL and cyclin D1 were measured by western blotting (left panel). Immunoblot analysis of Bcl-xL (middle panel) and cyclin D1 (right panel) with their relative band densities. GAPDH was used as a loading control. (**c**) Cells were stained by PI and annexin V to detect cell death and apoptosis by flow cytometry. The gating strategy is shown in the left two panels. The bar graphs show the percentage of annexin V^+^ cells (right panel). (**d**) Cell viability was tested by a CCK8 kit. (**e**) p-STAT6 was measured by western blotting (left panel). Immunoblot analysis of the ratio of pSTAT6/STAT6 in Nthy-ori 3-1 cells with their relative band densities (right panel). (**f**) Bcl-xL and cyclin D1 were measured by western blotting (left panel). Immunoblot analysis of Bcl-xL (middle panel) and cyclin D1 (right panel) with their relative band densities. GAPDH was used as a loading control. (**g**) Cells were stained with PI and annexin V to determine cell death and apoptosis by flow cytometry. The bar graphs show the percentages of annexin V^+^ cells (**h**) Cell viability was analysed by a CCK8 kit. The bar graph values are the means±S.E.M. of four independent experiments, each performed in duplicate. *P<0.05, **P<0.01 and ***P<0.001

We next stimulated the human TEC line Nthy-ori 3-1 with recombinant human IL-4 (rhIL-4). p-STAT6 was increased in a time-dependent manner, with a peak at 30 min (Figure 5a). The STAT6 downstream target Bcl-xL was also elevated in a time-dependent manner, and cyclin D1 increased with a peak at 24 h (Figure 5b). More importantly, as shown in Figures 5c and d, fewer than 2% of cells were stained with annexin V in the IL-4-treated group, which was a much lower percentage than that in the control group. Nthy-ori 3-1 cells stimulated by IL-4 exhibited increased cell viability in a time-dependent manner, both peaking at 48 h. These data suggest that IL-4 acts upstream of STAT6 in TECs and could be a crucial factor for TEC hyperplasia.

To determine whether STAT6 phosphorylation is essential for TEC hyperplasia, we used AS1517499, a widely used STAT6 inhibitor, to treat Nthy-ori 3-1 cells 30 min before IL-4 stimulation. As shown in Figures 5e and f, western blot examination revealed that AS1517499 (100 nM) blocked almost 80% of STAT6 phosphorylation induced by IL-4 and reduced cyclin D1 expression by 26% and Bcl-xL expression by 45%. STAT6 can prevent apoptosis and induce cell proliferation by regulating Bcl-xL and cyclin D1 expression in TECs, so we evaluated the viability and apoptosis of TECs. As shown in Figures 5g and h, Nthy-ori 3-1 cells stimulated by IL-4 exhibited less apoptosis and greater viability than the control cells. In contrast, in the AS1517499 plus IL-4-treated group, cell viability decreased significantly, as more than 6% of cells were stained with annexin V in the AS1517499 plus IL-4-treated group, which was a much higher percentage than that in the IL-4 group.

**Figure 6:**
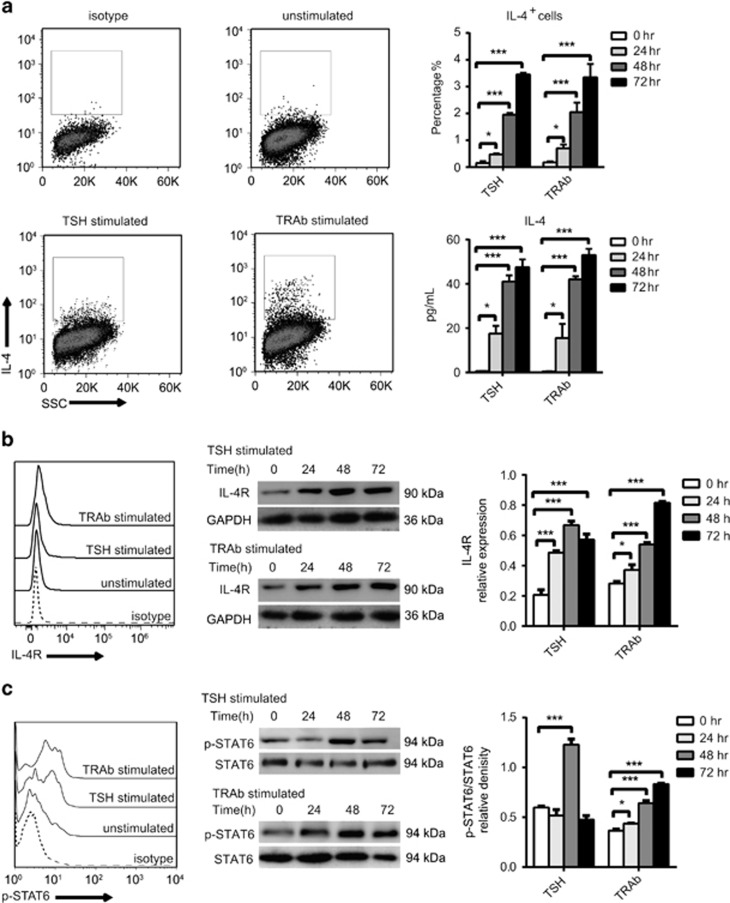
The level of IL-4 and IL-4R is activated by TRAb or TSH Nthy-ori 3-1 cells were stimulated by 1 μg/ml TRAb (M22) or TSH, and cells were harvested at different time points. (**a**) IL-4 was measured by FACS and ELISA (lower right panel). (**b**) IL-4 R was measured by FACS (left panel) and western blotting (right panel). Immunoblot analysis of IL-4 R, with the relative band densities, is shown; GAPDH was used as a loading control. (**c**) p-STAT6 was measured by FACS (left panel) and western blotting (right panel). Immunoblot analysis of the ratio of pSTAT6/STAT6 in Nthy-ori 3-1 cells with the relative band densities. The bar graph values are the means±S.E.M. of three independent experiments, each performed in duplicate. *P<0.05 and ***P<0.001

**Figure 7:**
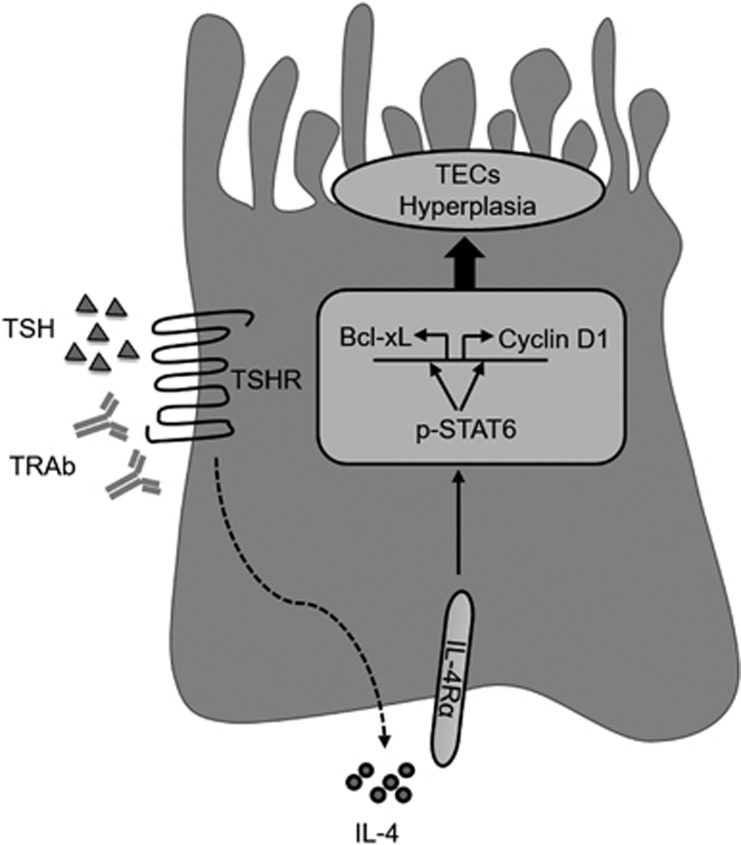
Scheme of the proposed mechanism of p-STAT6-mediated TEC hyperplasia in GD.

We next asked whether thyroid-stimulating hormone receptor (TSHR) stimulus initially triggered IL-4 production during GD. We used either TSH or TRAb to stimulate Nthy-ori 3-1 cells and detected the expression of the IL-4/IL-4 R/p-STAT6 axis. As we expected, the levels of IL-4, IL-4 R and p-STAT6 were upregulated after stimulation for 24 h or more (Figures 6a–c). These data suggest that TRAb triggers IL-4 production, and autocrine IL-4 subsequently elicits a positive feedback loop through a STAT6-Bcl-xL/cyclin D1 pathway, which induces hyperplasia of TECs (Figure 7).

### STAT6 blockade rescues GD *in vivo*

**Figure 8:**
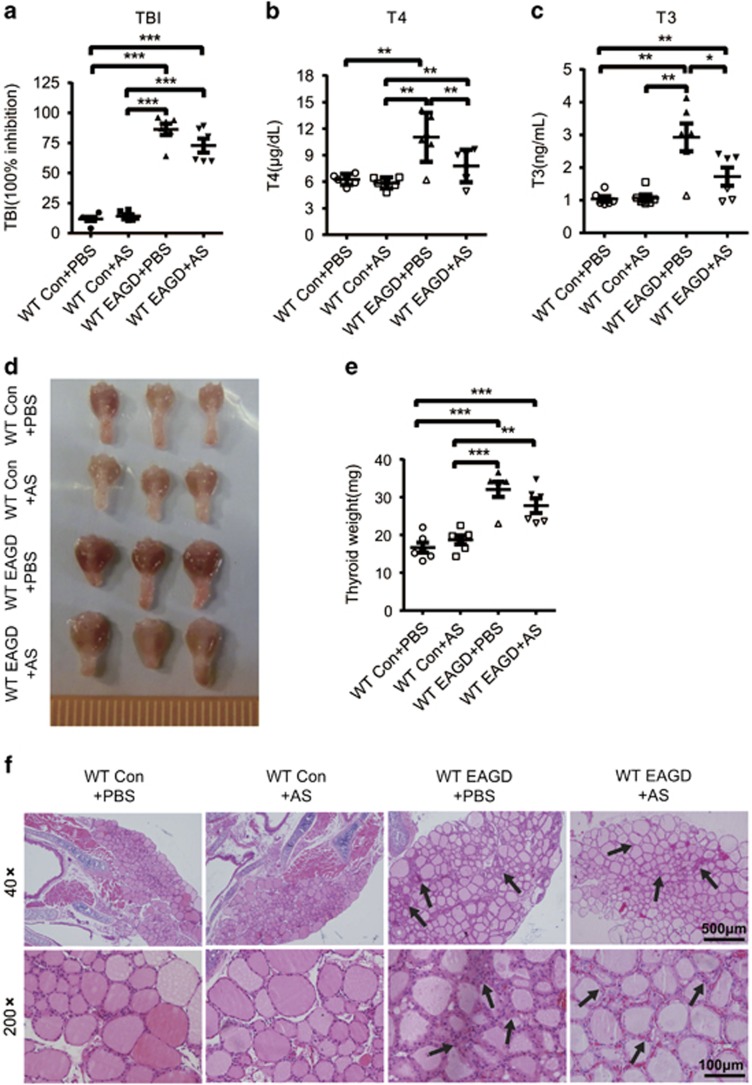
AS1517499 injection ameliorated disease in EAGD mice. Mice were injected with TSHR-289-expressing adenovirus and/or AS1517499 and were euthanized 3 weeks after the three injections. Blood and thyroid glands were obtained. Sera were analysed for TBI (**a**), T4 (**b**) and T3 (**c**). Data for individual mice are shown. (**d**) Thyroid glands were harvested for gross comparison and weight determination. Representative images show the appearance of thyroid glands from the control mice, EAGD mice and AS1517499-treated EAGD mice. (**e**) The weights of the thyroids were measured. The individual weights of the thyroids are shown with each point. (**f**) Representative histological sections of thyroid glands stained with H&E are shown for the control mice, EAGD mice and AS1517499-treated EAGD mice. Images are shown at × 40 and × 200 magnifications. An arrow indicates the location of thyrocyte hypercellularity in the histological images. The data shown are the means±S.E.M. of one experiment with six mice. Three independent experiments showed similar results. Scale bars (× 40): 500 μm; scale bars (× 200): 100 μm.*P<0.05, **P<0.01 and ***P<0.001.

Our data suggested that STAT6 could promote TEC hyperplasia in GD; therefore, we then aimed to determine whether STAT6 blockade could rescue GD by reducing TEC hyperplasia. Mice were treated with AS1517499 (10 mg/kg/d; dissolved in 20% dimethyl sulfoxide (DMSO)) or vehicle by intraperitoneal injection 1 h before Ad-TSHR-289 injection a total of four times (on days 21, 31, 42 and 52). We found that the AS1517499-treated mice exhibited significantly lower serum T3 and T4 levels compared with the DMSO-treated mice (Figures 8a–c). In addition, the size and weight of the thyroid glands from the AS1517499-treated mice were less than those of the thyroid glands from the DMSO-treated EAGD mice (Figures 8d and e). Histologically, the appearance of thyroid gland TECs and tissue changed less in the AS1517499-treated mice (Figure 8f). These results demonstrated that when EAGD mice have palpable signs, AS1517499 treatment after Ad-TSHR-289 injection was still capable of relieving disease symptoms in our experimental setting, indicating that a STAT6 blockade could relieve symptoms in GD patients.

We also performed exclusion experiments to eliminate the possibility that AS1517499 treatment could spontaneously induce GD or affect Th2 differentiation and Ig class switching. We compared the WT Con+PBS and WT Con+AS groups. As shown in Figures 6a–e, the WT Con+AS-treated mice exhibited similar T3 and T4 levels and histological features, as well as a similar thyroid appearance, to the WT Con+PBS-treated mice. In addition, WT Con+AS-treated mice expressed IL-4, IgG1 and IgG2a levels that were similar to WT Con+PBS-treated mice, as did WT EAGD+PBS versus WT EAGD+AS groups (Supplementary Figures 3B, D and F). These results indicate that AS1517499 treatment does not affect the function of the thyroid or GD-related cytokine production and antibody isotype switching.

## Discussion

GD is an organ-specific autoimmune disease. TECs from GD patients grow vigorously and produce T3 and T4 without restraint, resulting in a clinical outcome of hyperthyroidism.^25, 26^ Current treatments for GD and the related hyperthyroidism are inadequate because the treatments, including radioactive iodine ablation, thyroid surgery and drugs for treating thyrotoxicosis (for example, propylthiouracil), are invasive or only target the signs and symptoms of the disease rather than the pathophysiology.^3^ The pathogenesis of GD remains unclear. It has been shown that TRAb targeting of TSHR could result in TEC hyperplasia and, consequently, lead to hyperthyroidism.^27, 28^ However, TRAb depletion treatment using rituximab is very offensive with aggravating ulcerative colitis and similar diseases.^7, 8^ Thus, we believe that there must have been another crucial factor that promotes TEC hyperplasia. Our study demonstrates that autocrine IL-4 activity in TECs promotes TEC hyperplasia by activating p-STAT6 and its downstream targets cyclin D1 and Bcl-xL. Moreover, blockade of STAT6 phosphorylation by AS after GD onset is able to rescue the disease, which indicates that, as a targeted biological therapy, AS 1517499(AS) could serve as a new treatment for GD. To our knowledge, this study is the first to demonstrate that increased p-STAT6 in TECs could be an essential factor involved in TEC hyperplasia during GD, complementing the conventional knowledge that stimulation of the TSHR and releasing T3, T4 is the only way to promote TEC hyperplasia and, thus, GD.

As discussed previously, the exact cause of TEC hyperplasia in GD remains unclear. Immunoregulatory and thyroid-specific genes, such as HLA, CTLA-4, TSHR and TG, are thought to contribute to the aetiology of GD and TEC hyperplasia.^29^ However, drugs that target these genes are either impractical or inefficient. STAT6 is an important transcription factor in epithelial cell growth, and it is related to various autoimmune diseases and inflammatory conditions. According to Khaled *et al*, luminal mammary epithelial cell development is promoted by cytokine/STAT6.^30^ Thus, important questions have emerged regarding the function of STAT6 in TECs and the consequences of its activity. We hypothesize that p-STAT6 is highly induced in TECs in GD and is required for promoting TEC hyperplasia and aggravating disease symptoms. Rosen *et al* reported that STAT6 activated in colon epithelial cells results in intestinal disorders and colitis, but they did not demonstrate whether STAT6 is related to epithelial cell hyperplasia.^13^ Our results demonstrate that STAT6 is responsible for TEC hyperplasia in GD, as hyperplastic TECs from both GD patients and EAGD mice highly expressed p-STAT6, and STAT6 deficiency reduced the disordered growth of TECs and rescued GD. It is noteworthy that when we used STAT6^−/−^ mice to generate EAGD mice, the serum T3 and T4 levels decreased and the thyromegaly was diminished compared with the WT EAGD group. These findings suggest that STAT6^−/−^ relieved the symptoms of GD and rescued the hyperthyroidism. However, according to our TRAb detection, the TSHR-specific antibody was not altered in the STAT6^−/−^ GD group, consistent with the previous finding that STAT6 may serve as a cell activator in GD.^31^ There are multiple causes of cell hyperplasia. However, at the cellular level, unrestrained proliferation and rare apoptosis are the major reasons for cell hyperplasia. We showed that cell apoptosis was inhibited in TECs from WT EAGD mice, while STAT6 deficiency rescued this disorder. Similarly, cell proliferation was vigorous in TECs from WT EAGD mice, while STAT6 deficiency normalized this phenotype. We also revealed that the STAT6 target Bcl-xL was responsible for inhibiting apoptosis and that cyclin-D1 was a key factor in cell proliferation. Other proteins, such as Bcl-2, Bax, Bad, p21, p27^kip1^ and PCNA, are likely to be important contributors to cell growth,^19, 21, 22, 24, 32, 33, 34, 35, 36^ but our experiments showed that the expression of these genes did not change in TECs from EAGD mice or patients (data not shown). Notably, the cytokines responsible for activating p-STAT6 are diverse. IL-4, IL-13, IL-22, IL-25 and IL-33 are important in activating p-STAT6.^14, 15, 16, 17, 37^ We determined the levels of these cytokines from thyroid tissue homogenates and supernatants, and only IL-4 and IL-4Rα were significantly changed in GD, which was consistent with a previous study showing that IL-4 deficiency could alleviate EAGD.^38^ Previous studies indicated that interstitial lymphocytic infiltration is not involved in the EAGD mouse model; thus, we asked whether the IL-4 was derived from draining lymph nodes.^39^ Unexpectedly, IHC and flow cytometry (Figure 4d and data not shown) showed that IL-4, which activated p-STAT6, was not derived from lymphocytes but from TECs. We were initially surprised by the results. However, after repeating the experiments several times, we confirmed the results. In addition, we found similar phenomena reported in the literature. For example, autocrine production of IL-4 and IL-10 regulates thyroid epithelial cancer cell survival, growth and resistance to chemotherapy.^40, 41^ In our system, lymphocytes rarely infiltrated the thyroid of EAGD mice, and IHC staining demonstrated that TECs could produce IL-4. Furthermore, this result was later confirmed by FACS analyses. Thus, we believe that TECs could be the source of IL-4 that contributes to the pathogenic mechanism of GD. We next explored the factor that triggers IL-4 production. According to some studies, human fibrocytes express high levels of TSHR when treated with TSH or a TSHR-activating antibody (TRAb), such as M22. These cells then produce high levels of proinflammatory cytokines, such as IL-6, IL-8 and TNF-alpha.^42, 43^ Thus, it is possible that TSH and/or TRAb stimulation could contribute to the triggering of IL-4 production during GD. We stimulated the TEC line Nthy-ori 3-1 with TSH or TRAb (M22) *in vitro*, and we observed that the expression of IL-4, IL-4Rα and p-STAT6 was upregulated after the stimulation. This finding demonstrated that TRAbs could be the initial trigger for IL-4 production during GD, and IL-4 consequently promoted STAT6 phosphorylation, which contributed to TEC hyperplasia during GD. This is an important pathway in addition to the conventional TRAb-TSHR-T3/T4 mechanism.

AS1517499 is used to inhibit the phosphorylation of STAT6 with high specificity, and it often serves as a biological therapy for diseases such as intestinal ischaemia/reperfusion (I/R) injury and bronchial hypercontractility.^44, 45^ The exact mechanisms of AS1517499 inhibiting STAT6 are still unclear. Nonetheless, according to reports, AS1517499 (UNII-2H31HOT08T), soluble in purified DMSO, is a potent and selective STAT6 inhibitor with an IC50 of 21 nM.^46^ This compound is a novel p-STAT6 inhibitor that was synthesized based on the structure of a previously reported STAT6 inhibitor, TMC-264, discovered from the fungus *Phoma*.^47, 48^ We used AS1517499 1 h before the second immunization with Ad-TSHR289, when the mice already had some symptoms of GD, such as mild thyromegaly and slight but significant increases in T3 and T4 (data not shown). As we demonstrated previously, AS1517499 successfully rescued GD even after the disease was initiated. We then observed the overall physiological condition of the mice and evaluated liver and kidney function, and we did not find abnormal signs (data not shown). Together, our data demonstrate that p-STAT6 inhibition treatment could be a potent therapy for GD.

In conclusion, our study demonstrates that in addition to the well-known TRAb pathway, the TEC-derived IL-4-STAT6-Bcl-xL/cyclin D1 pathway may contribute to the pathogenesis of GD by stimulating unrestrained TEC hyperplasia. Our findings suggest that p-STAT6 inhibition treatment is critical for the rescue of GD and could be more specific with fewer side effects than traditional GD treatments. We hope that our study provides a new approach for studying the aetiology of GD and diagnosing and treating it in the clinic.

## Materials and msethods

### Human samples

Thyroid tissues from GD patients (*n*=10) were obtained at the time of thyroidectomy, and normal thyroid samples (*n*=8) were obtained from unaffected glands of patients undergoing parathyroidectomy in accordance with the established diagnosis on the basis of commonly accepted clinical, laboratory and histological criteria. The study was approved by the ethics committee of Shanghai Fifth People's Hospital (approval number 030, 2012).

### Construction of Ad-TSHR-289

Adenoviruses containing TSHR amino acid residues 1–289 (Ad-TSHR-289) and control adenovirus (Ad-Con) were constructed and purified as described.^39^ Briefly, Ad-TSHR-289 and Ad-Con were propagated in HEK-293 cells, and the concentration of viral particles was determined by measuring the absorbance at 260 nm. The viruses used in the study were all stored in aliquots at −80 °C.

### EAGD mouse model induction

Wild-type (WT) BALB/c mice were obtained from the Shanghai SLAC Laboratory Animal Co., Ltd. (Shanghai, China). STAT6^−/−^ mice (BALB/c background) were kindly provided by Dr Rui He (Department of Immunology, Fudan University, Shanghai, China). All mice were maintained in the animal facility of Fudan University under specific pathogen-free barrier conditions. Female mice aged 6–7 weeks were injected intramuscularly with Ad-TSHR289 or Ad-Con (5 × 10^10^ particles in 50 μl of phosphate-buffered saline (PBS)) as described previously.^39^ Mice were injected three times at 3-week intervals and were euthanized 4 weeks after the third injection to obtain blood and thyroid glands. The blood was collected and centrifuged at 1500 × g for 10 min, and the serum was stored at −80 °C until hormone analysis. All experimental procedures on animals were carried out in compliance with the Guide for the Care and Use of Laboratory Animals.

### TSH-binding inhibition assay

TBI was measured using enzyme-linked immunosorbent assay (ELISA) kits (ElisaRSRTM TRAb 2nd Generation kit, RSR Limited, Cardiff, UK) according to the manufacturer's protocol. Briefly, 75 μl serum aliquots of mice were incubated with immobilized TSHR coated onto ELISA plate wells. TSH-biotin was added, and the amount of TSH-biotin bound to the plate was determined by the addition of streptavidin peroxidase (SA-POD), which binds specifically to biotin. This assay measures the ability of TRAb in the serum to inhibit the binding of TSH-biotin to TSHR. The TBI values were calculated using the following formula: 100 × (1-test sample absorbance at 450 nm/negative control absorbance at 450 nm). Values greater than the normal range (mean±2  S.D. of TBI activity in WT Con mouse serum) were considered positive.

### Analysis of total T4 and T3

The total serum T3 and T4 levels were measured with a commercially available kit (Roche Applied Science, Upper Bavaria, Germany). The normal range was defined as the mean±2 S.D. of WT Con mice serum.

### Thyroid weight and histology

Thyroid tissues were weighed and fixed in 10% formalin. Tissues were embedded in paraffin; then, 5-μm thick sections were prepared and stained with haematoxylin and eosin.

### Nthy-ori 3-1 cell culture and sample collection

Considering the limited numbers of primary TECs that can be collected, Nthy-ori 3-1 cells were used as a model for studying signal transduction pathways. Nthy-ori 3-1, a normal human thyroid follicular epithelial cell line, was purchased from the Guangzhou Jenniobio Biotechnology Co., Ltd. (Guangzhou, China). Nthy-ori 3-1 cells were cultured at 37 °C in a 5% CO_2_ humidified incubator in RPMI 1640 (Gibco, Portland, OR, USA) supplemented with 10% foetal bovine serum (Gibco, Portland, OR, USA), 2 mmol/L glutamine (Gibco, Portland, OR, USA), 100 IU/mL penicillin and 100 mg/mL streptomycin sulfate.

### Nthy-ori 3-1 cell viability assay

Nthy-ori 3-1 cell viability was measured using a Cell Counting Kit-8 (CCK-8; Dojindo, Kunamoto, Japan) based on a redox assay similar to the 3-(4,5)-dimethylthiahiazo (-z-y1)-3,5-di- phenytetrazoliumromide assay.

### Nthy-ori 3-1 cell inhibition assay

The STAT6 inhibitor AS1517499 (AXON Medchem BV, The Netherlands) was dissolved in DMSO. Nthy-ori 3-1 cells were seeded into 6- and 96-well plates. When 60–70% confluence was observed, cells were cultured without serum for 24 h before the addition of recombinant human IL-4 (PeproTech EC, Ltd., London, UK). AS1517499 or its vehicle, 0.3% DMSO, was added 30 min before the addition of IL-4 (50 ng/ml). At the indicated time after the IL-4 treatment, cells were collected and analysed by quantitative reverse transcription-polymerase chain reaction (qRT-PCR), western blot, CCK8 assay and FACS analysis.

### Nthy-ori 3-1 cell stimulation assay

TSH (Sigma-Aldrich, St Louis, MO, USA) and TRAb (RSR-TSHR hMAB M22, Cardiff, UK) were dissolved in water or 1% BSA. Nthy-ori 3-1 cells were seeded into 24-well plates. When 60–70% confluence was observed, cells were cultured without serum for 24 h before the addition of 1 μg/ml TSH or TRAb. At the indicated time after the TSH or TRAb stimulation, cells were collected and assayed by FACS, western blot and ELISA.

### STAT6 inhibitor treatment of EAGD mice

STAT6 inhibitor treatment was performed as described previously.^44, 45^ Briefly, 6-week-old female BALB/c mice were randomly assigned to one of four experimental groups (*n*=6 mice/group). The mice were injected intramuscularly with Ad-TSHR289 or Ad-Con on days 0, 21 and 42 to generate the EAGD mouse model. The groups were treated with either AS1517499 (10 mg/kg/d; dissolved in 20% DMSO) or vehicle by intraperitoneal injection a total of four times on days 21, 31, 42 and 52. If the inhibitor treatment was administered on the same day as the adenovirus injection, the intraperitoneal injection with AS1517499 or vehicle was performed 1 h before the Ad-TSHR289 or Ad-Con injection (days 21 and 42). Three weeks after the last adenovirus injection, the mice were sacrificed to obtain blood and thyroid glands.

### Immunohistochemistry

The thyroids were fixed in paraformaldehyde, embedded in paraffin, and sectioned to a thickness of 5 μm for immunohistochemical analysis. After deparaffinization, rehydration and repair in citrate buffer (Beyotime Biotech Inc., Shanghai, China), and elimination of endogenous peroxidase with 3% H_2_O_2_, sections were incubated with 3% BSA for 1 h at room temperature. Sections were stained with rabbit monoclonal antibody against phospho-STAT6, Ki67, IL-4 R (all from Abcam, Inc., Cambridge, UK), or IL-4 (Thermo Fisher Scientific, PA5-25165, Waltham, MA, USA) overnight at 4 °C. The secondary antibody, HRP-labelled anti-rabbit immunoglobulin (Gene Tech, Shanghai, China), was applied for 1 h at room temperature. Sections were washed in PBS, followed by diaminobenzidine substrate for 1 min and haematoxylin for 1 min. Finally, sections were rinsed with water, dehydrated, cleared and mounted with neutral balsam. Immunohistochemical images were obtained with a Leica microscope camera and an Olympus microscope camera system. Six fields at × 400 magnification were selected randomly to quantify the number of p-STAT6-positive, Ki67-positive, IL-4-positive and IL-4 R-positive epithelial cells per high-powered field (HPF).

### Apoptosis assays of TECs

The apoptotic TECs were stained with a terminal deoxynucleotidyl transferase-mediated deoxyuridine triphosphate nick-end labelling (TUNEL) assay (Apoptosis Detection Kit, Vazyme, China) according to the manufacturer's protocol and were analysed by the Olympus microscope camera system. Six fields at × 400 magnification were selected randomly to calculate the number of TUNEL-positive cells in every HPF. The apoptosis of Nthy-ori 3-1 cells was analysed by FACS analysis. Adherent cells were suspended in ice-cold PBS containing 0.5% bovine serum albumin. FcR II/III receptors were labelled before staining with specific antibody. Antibodies against annexin V and propidium iodide (GE Healthcare, Little Chalfont, UK) were used for flow cytometry according to the manufacturer's instructions. Cells were analysed in a CyAn ADP Analyser (Beckman, Coulter, Inc., Carlsbad, CA, USA), and data were analysed using FlowJo software (Tree Star, Ashland, OR, USA).

### Western blot analysis

Thyroid proteins and Nthy-ori 3-1 cells were prepared with radio-immunoprecipitation assay lysis buffer for western blot analysis, mixed with 5X SDS loading buffer, and denatured at 100 °C for 10 min. Proteins were separated by 10% sodium dodecyl sulfate polyacrylamide gel electrophoresis (SDS-PAGE) (Beyotime Biotech Inc., Shanghai, China) and transferred electrophoretically onto a polyvinylidene difluoride membrane (Merck Millipore, Darmstadt, Germany). After the membranes were blocked with 5% non-fat milk for 2 h at room temperature, they were incubated overnight at 4 °C with anti-p-STAT6, anti-STAT6, anti-cyclin D1, anti-IL-4 R (all from Abcam, Inc., Cambridge, UK), anti Bcl-xL (Cell Signaling Technology, Inc., 2764, Danvers, MA, USA), and anti-GAPDH (Kangchen Bio-tech Inc., KC-5G5, Shanghai, China) antibodies. After the membranes were rinsed, they were incubated with the HRP-conjugated secondary antibody for 2 h at room temperature. The immune-reactive protein was detected by an enhanced chemiluminescence western blot detection system (Thermo Fisher Scientific, Waltham, MA, USA). The densitometric analysis of immunoblots was performed using ImageJ software.

### Real-time reverse transcriptase-PCR analysis

Total RNA was extracted with TRIzol® reagent (Invitrogen Life Sciences, Carlsbad, CA, USA) according to the manufacturer's protocol and reverse-transcribed into complementary DNA (Takara Biotechnology Co., Ltd, Dalian, China). Real-time PCR was performed in the ABI 7500 thermocycler using 1 μl of cDNA, primers and SYBR® Green Real-time PCR Master Mix (Toyobo Co., Ltd., Osaka, Japan). The primer sequences are listed in Table 1. Thermocycling was performed as follows: initial denaturation at 95 °C for 30 s; 45 cycles of denaturing at 95 °C for 5 s and elongating at 60 °C for 15 s. The ΔΔCt calculation method was used to compute the relative gene expression compared with *β*-actin. All primers were synthesized and purified by Shanghai Sangon Biotechnology Co. Ltd. (Shanghai, China).

### Statistical analysis

Data were analysed using SPSS 15.0 (SPSS) and PRISM 6.0 (GraphPad Software Inc., La Jolla, CA, USA) software and were presented as the mean±standard error of the mean (S.E.M.). Fisher's exact test was used to determine the significance of differences between the number of mice in a group positive or negative for a particular parameter. Student's unpaired *t*-test or unpaired *t*-test with Welch's correction was used to determine significant differences, one-way analysis of variance was used to analyse multiple groups, and Pearson's correlation coefficient was used to analyse the correlation between groups. Multiple group comparisons were performed using one-way analysis of variance or Kruskal–Wallis test, followed by Bonferroni Correction or Mann–Whitney to compare two individual groups. *P* values<0.05 were considered statistically significant.

## Figures and Tables

**Table 1:** Primer information

**Primer**	**Sequence(5′-3′)**
Bcl-xL F (Forward)	AACATCCCAGCTTCACATAACCC C
Bcl-xL R (Reverse)	GCGACCCCAGTTTACTCCATCC
Cyclin D1 F (Forward)	GCGTACCCTGACACCAATCTC
Cyclin D1 R (Reverse)	ACTTGAAGTAAGATACGGAGGGC
IL-4 F (Forward)	ACAGGAGAAGGGACGCCATGCACGGAGAT
IL-4 R (Reverse)	GCGAAGCACCTTGGAAGCCCTACAGACGAG
IL-10 F (Forward)	GCTCTTACTGACTGGCATGAG
IL-10 R (Reverse)	CGCAGCTCTAGGAGCATGTG
IL-13 F (Forward)	ACTGCCTGCCTAGTGCTCAG
IL-13 R (Reverse)	GCTGGACTCACGGTCTTGCT
Il-22 F(Forward)	ATGAGTTTTTCCCTTATGGGGAC
IL-22 R (Reverse)	GCTGGAAGTTGGACACCTCAA
IL-25 F (Forward)	GCTATGAGTTGGACAGGGACTTGAATCG
IL-25 R (Reverse)	GGTTGTGGTAAAGTGGGACGGAGTTG
IL-33 F (Forward)	AGACCAAGAGCAAGACCAGGTGCTACTACG
IL-33 R (Reverse)	GCTTCTTCCCATCCACACCGTCGCCTGAT
IL-4Rα F (Forward)	CTGGCTGTGGCTGCTGCTACGATGA
IL-4Rα R (Reverse)	GCTGACATGAGGTTGGCTTCTGGTGGTATT
IL-13Rα1 F (Forward)	GGCGAGCTGTTGGTGCTGCTACTGT
IL-13Rα1 R (Reverse)	CGCTCAAATTCGTCACAGGTGGCTGAACTT
β-actin F (Forward)	CTGTCGAGTCGCGTCCACC
β-actin R (Reverse)	CATGCCGGAGCCGTTGTCG
