# Supplementary material for: STAT6 deficiency ameliorates Graves' disease severity by suppressing thyroid epithelial cell hyperplasia
Source: Cell Death Dis. 2016 Dec 1;7(12):e2506–. doi: 10.1038/cddis.2016.398 (PMC5260978; doi:10.1038/cddis.2016.398)
Supplement: Supplementary Figures [file cddis2016398x1.docx]

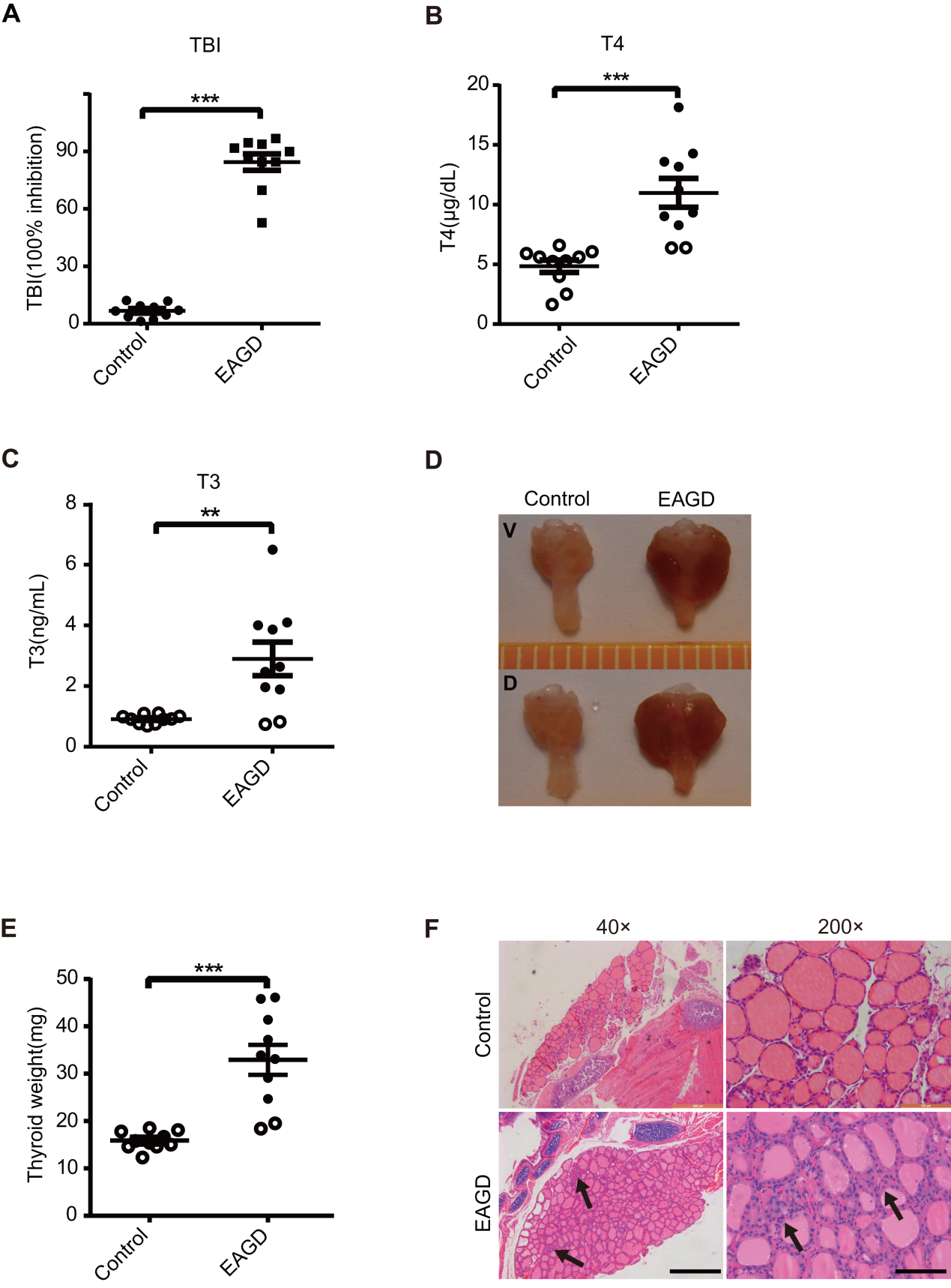


**Supplementary Figure 1: Induction of EAGD mice** Mice were injected intramuscularly with TSHR-289-expressing adenovirus Ad-TSHR289 or with control adenovirus Ad-Con three times at three-week intervals. Mice were euthanized 4 weeks after the third injection, and blood and thyroid glands were obtained. Sera were analyzed for TBI (A), T4 (B), and T3 (C). Data for individual mice are shown. TBI was essentially undetectable in control mice but significantly increased in EAGD mice. Similarly, T3 and T4 levels were elevated in the EAGD group. (D) Upon sacrifice of the mice, thyroid glands were harvested for gross comparison and weight determination. Representative images show the appearance of thyroid glands from the control and EAGD groups. (E) The weights of thyroids were measured (left panel). Individual thyroid weights are shown at each point. The macroscopic ﬁndings showed that the thyroid glands of EAGD mice had marked hypertrophy. Statistical analysis conﬁrmed that the mean weight of the thyroid glands from the EAGD mice was 35 mg *vs* 15 mg in the control mice. (F) Representative histological sections of thyroid glands stained with hematoxylin and eosin (H&E) are shown for the control and EAGD groups. Images are shown at 40× and 200× magnification. An asterisk indicates the position of TEC H/P in the histological images. The data shown are the means ± SEM of one experiment with ten mice. Three repetitions showed similar results. Scale bars(40×): 500μm； Scale bars(200×)： 100μm. *P<0.05, **P<0.01, and ***P<0.001.


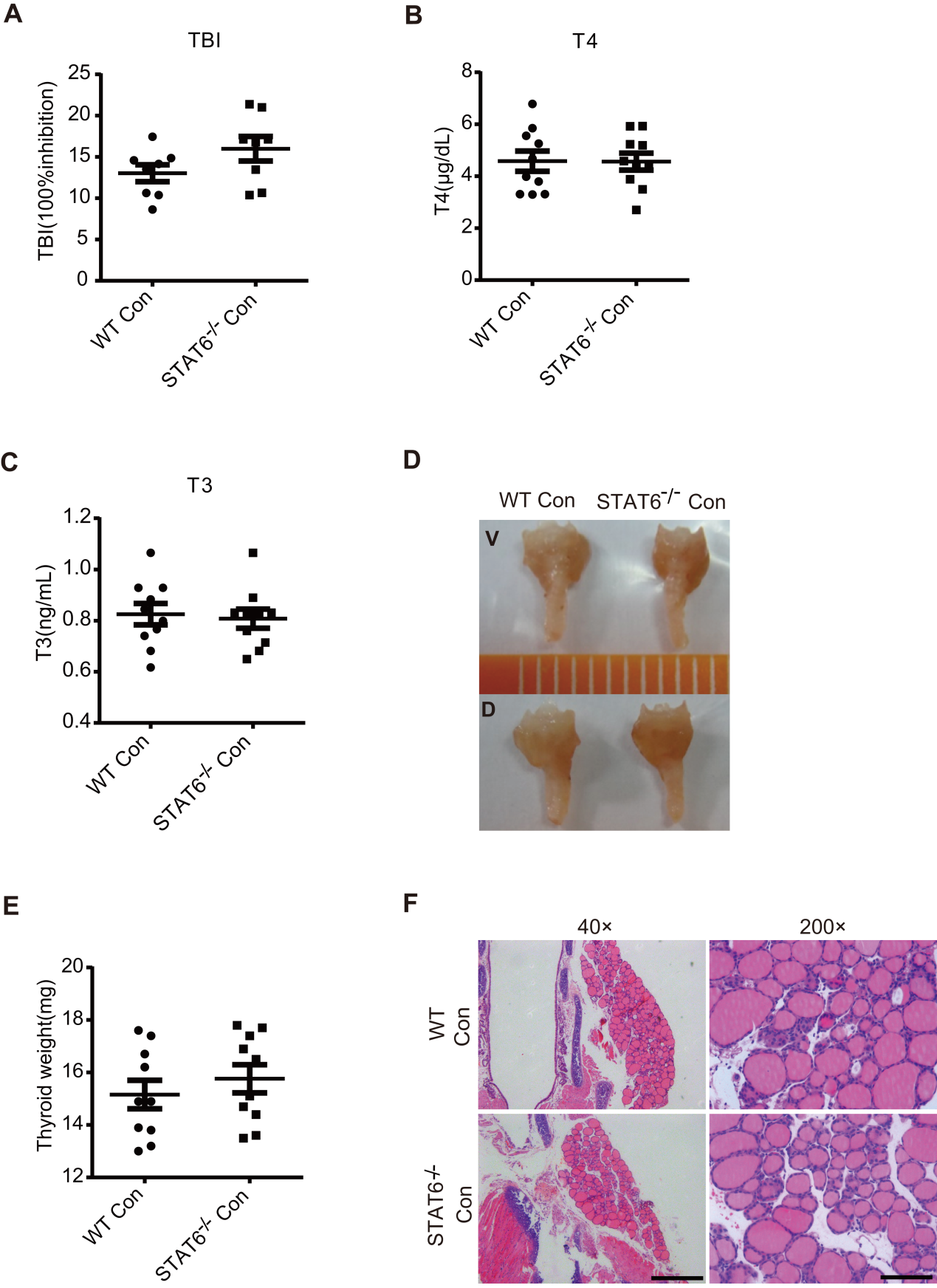


**Supplementary Figure 2: STAT6^-/-^ mice show normal thyroid function** WT and STAT6^-/-^ mice were euthanized, and their blood and thyroid glands were obtained. Sera were analyzed for TBI (A), T4 (B), and T3 (C). Data for individual mice are shown. (D) Thyroid glands were harvested for gross comparison and weight determination. Representative images show the appearance of thyroid glands from WT and STAT6^-/-^ mice. (E) The weights of thyroids were measured (left panel). Individual weights of thyroids are shown with each point. (F) Representative histological sections of thyroid glands stained with H&E are shown for the WT and STAT6^-/-^ mice. Images are shown at 40× and 200× magnification. The data shown are the means ± SEM of one experiment with ten mice. Three independent experiments showed similar results. Scale bars(40×): 500μm； Scale bars(200×)： 100μm.


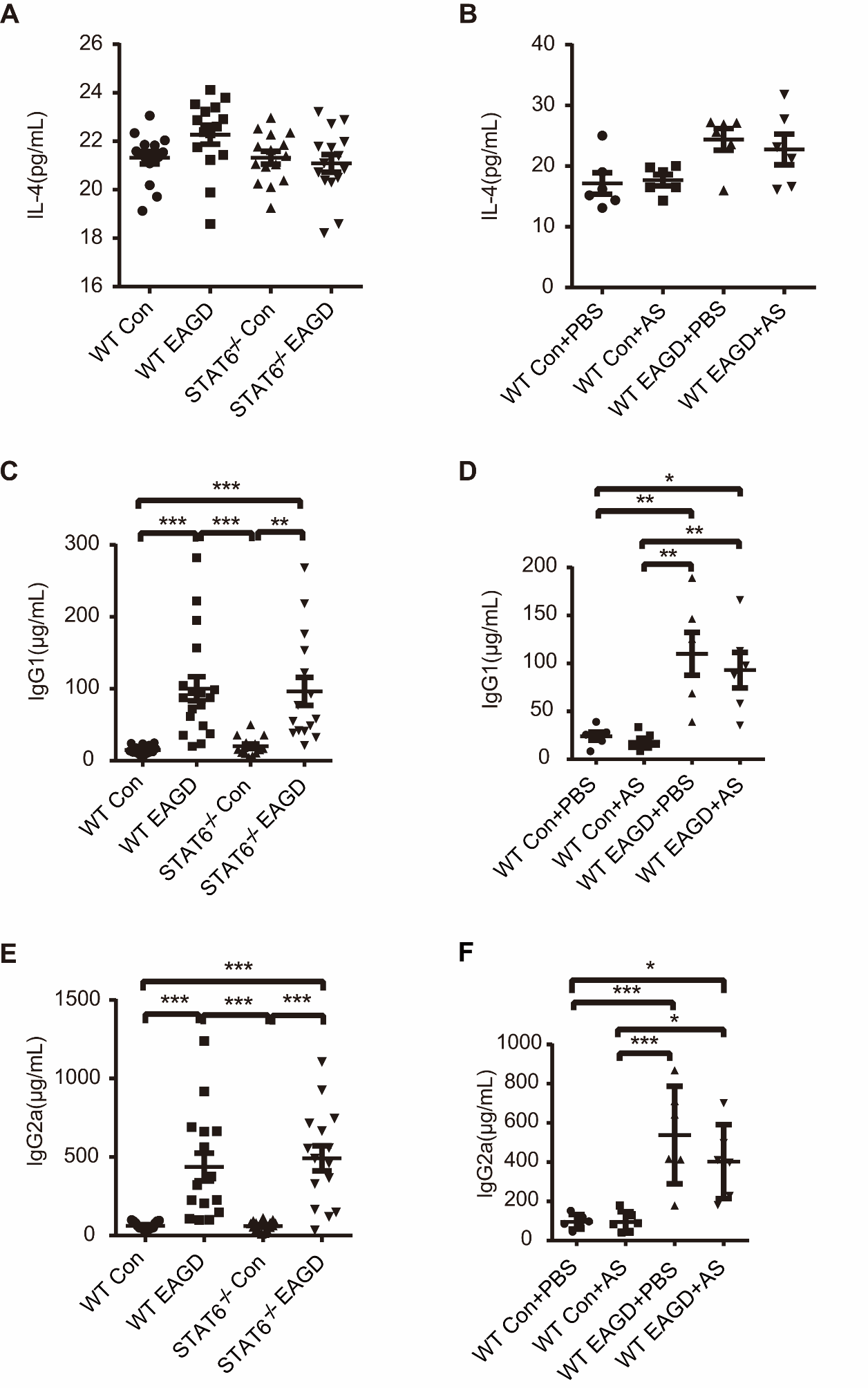


**Supplementary Figure 3: The expression of IL-4, IgG1 and IgG2a in the serum of control and EAGD mice** Serum were obtained from control and EAGD mice 4 weeks after three injections. ELISA analysis of IL-4 (A,B), IgG1 (C,D), and IgG2a (E,F) in Serum. The data shown are the means ± SEM of one experiment with ten mice. Three replicates showed similar results. *P<0.05, **P<0.01, and ***P<0.001.


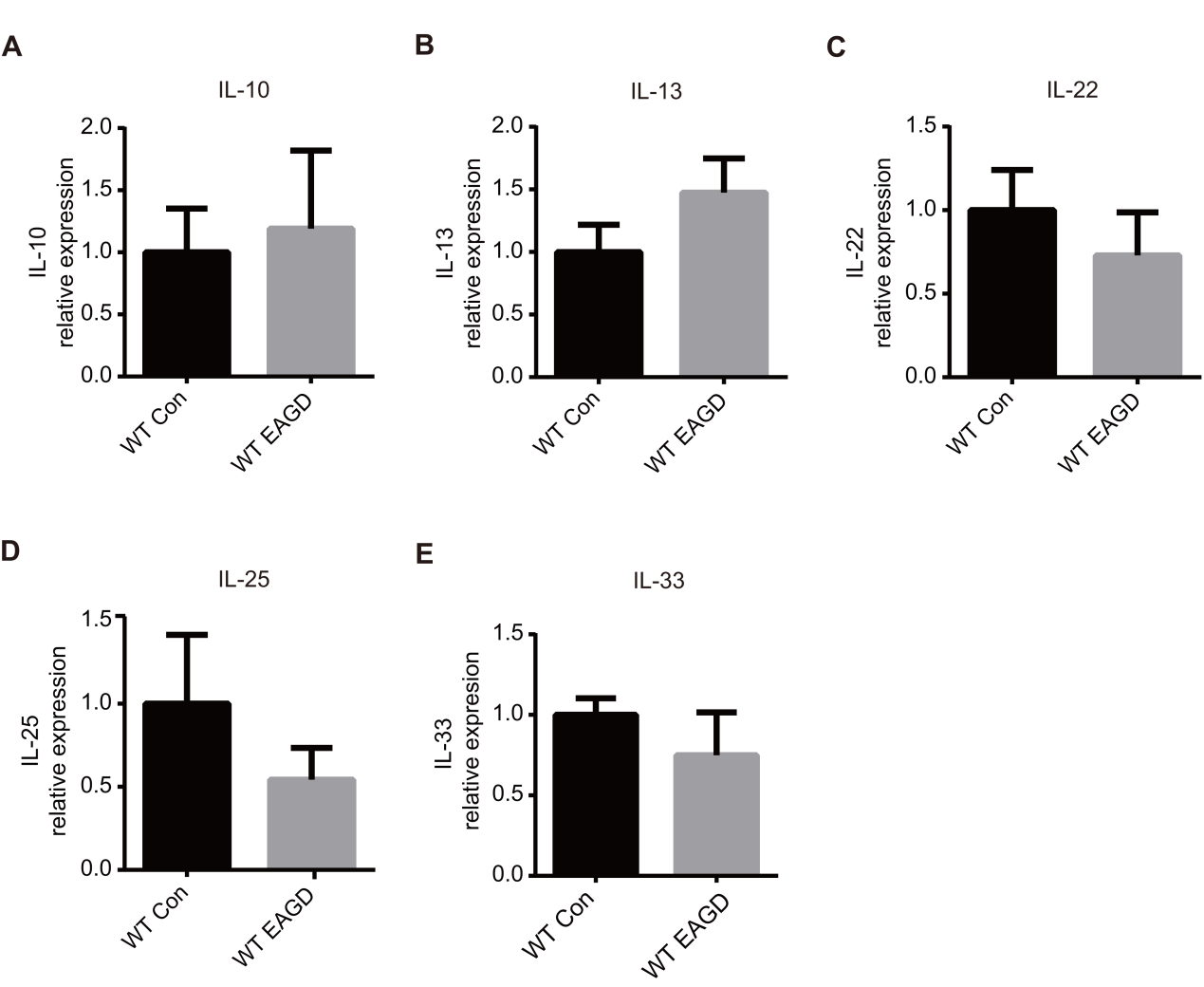


**Supplementary Figure 4: The mRNA levels of IL-10, IL-13, IL-22, IL-25, and IL-33 in the thyroids of control and EAGD mice.** Thyroid glands were obtained from control and EAGD mice 4 weeks after three injections. Quantitative real-time RT-PCR analysis of IL-10 (A), IL-13 (B), IL-22 (C), IL-25 (D), and IL-33 (E) in fresh thyroid glands. The data shown are the means ± SEM of one experiment with ten mice. Three replicates showed similar results.
